# Personal and Neighborhood Attributes Associated with Cervical and Colorectal Cancer Screening in an Urban African American Population

**DOI:** 10.5888/pcd16.190030

**Published:** 2019-08-29

**Authors:** James W Buehler, Juan C. Castro, Suzanne Cohen, Yuzhe Zhao, Steven Melly, Kari Moore

**Affiliations:** 1 Urban Health Collaborative and Department of Health Management and Policy, Dornsife School of Public Health, Drexel University, Philadelphia, Pennsylvania; 2 Family Practice and Counseling Network, Philadelphia, Pennsylvania; 3 Health Federation of Philadelphia, Philadelphia, Pennsylvania; 4 Urban Health Collaborative, Dornsife School of Public Health, Drexel University, Philadelphia, Pennsylvania

## Abstract

**Introduction:**

Assessing individual social determinants of health in primary care might be complemented by consideration of population attributes in patients’ neighborhoods. We studied associations between cervical and colorectal cancer screening and neighborhood attributes among an African American population in Philadelphia.

**Methods:**

We abstracted demographic and cancer screening information from records of patients seen during 2006 at 3 federally qualified health centers and characterized patients’ census tracts of residence by using census, survey, and other data to define population metrics for poverty, racial segregation, educational attainment, social capital, neighborhood safety, and violent crime. We used generalized estimating equations to obtain adjusted relative risks of screening associated with individual and census tract attributes.

**Results:**

Among 1,708 patients for whom colorectal cancer screening was recommended, screening was up to date for 41%, and among 4,995 women for whom cervical cancer screening was recommended, screening was up to date for 75%. After controlling for age, sex (for colorectal cancer screening), insurance coverage, and clinic site, people living in the most racially segregated neighborhoods were nearly 10% more likely than others to be unscreened for colorectal cancer. Other census tract population attributes were not associated with differences in screening levels for either cancer.

**Conclusions:**

The association between lower rates of colorectal cancer screening and neighborhood racial segregation is consistent with known barriers to colonoscopy among African Americans combined with effects of segregation on health-related behaviors. Recognition of the association between segregation and lower colorectal cancer screening rates might be useful in informing and targeting community outreach to improve screening.

**Summary**

**What is already known on this topic?**

Screening rates for colorectal and cervical cancer vary by patient attributes that reflect social determinants of health.

**What is added by this report?**

Among an urban African American population receiving primary care, census tract population attributes were not associated with screening levels after taking personal characteristics into account, except that those living in more racially segregated areas were less likely to be screened for colorectal cancer.

**What are the implications for public health practice?**

Although neighborhood attributes are unlikely to be a surrogate for individual-level screening for social health determinants, efforts to improve colorectal cancer screening among African American populations might be enhanced by outreach tailored to areas with high rates of racial segregation.

## Introduction

Spurred by a mix of policy and insurance initiatives, health care providers in the United States are developing strategies to address social determinants of health among their patients, with the aim of reducing health risks and improving health outcomes (1). Community health centers supported by the Health Resources and Services Administration (HRSA) have been at the forefront of efforts to promote screening for risks associated with a person’s social environment and refer patients to community services (2,3). Despite the promise of these efforts, many primary care providers feel unprepared to address health concerns related to patients’ social circumstances, making them reluctant to adopt new screening tools (4), and optimal approaches for using electronic health records (EHRs) to characterize social determinants of health have yet to be established (5). A possible complement to screening patients directly for social health risks is the use of geographically defined measures, which might have the added benefit of being immune from the limits of self-reported social indexes. Social determinants of health cluster in neighborhoods (6,7), and neighborhood indicators might aid in identifying patients who are more or less likely to experience the intended benefits of health care. For priority clinical preventive services, such as cancer screening, knowledge of the attributes of patients’ neighborhoods might illuminate variations in adherence to screening recommendations. Although population surveys have identified associations between respondents’ characterization of their neighborhoods and cancer screening (8,9), studies using place-based measures have yielded inconsistent results (10).

We examined associations between place-based neighborhood attributes and screening for cervical and colorectal cancer among African American patients receiving primary care within a network of federally qualified health centers (FQHCs) in Philadelphia.

## Methods

**Study population. **The study population comprised Philadelphia residents with 1 or more visits during 2016 to any of 3 FQHCs operated by the Family Practice and Counseling Network (FPCN) (Figure). These clinics are recognized as “patient-centered medical home” providers by the National Committee on Quality Assurance and offer primary care, behavioral health, dental, and social services for medically underserved populations (11,12). Since 2014, the FPCN has participated in a regional HRSA-funded project to strengthen FQHCs’ uses of health information technology (13), including uses of EHRs to improve the delivery of recommended preventive services. FPCN serves patients who reside in low-income neighborhoods. Among overall populations in the census tracts where patients recommended to receive colorectal cancer screening (14) resided, the median proportion living in households with incomes below the federal poverty threshold was 28% (interquartile range = 16%–38%) versus 10% (interquartile range = 7%–17%) for all other census tracts in Philadelphia, a pattern that was similar for cervical cancer screening. Among patients for whom colorectal or cervical cancer screening was recommended (14), their race/ethnicity was classified as non-Hispanic African American or black for 85% and 87%, respectively. Because the number of patients in other racial/ethnic groups was too small for statistically meaningful comparisons, we excluded them. Cervical cancer screening was conducted at FPCN clinics as part of routine care; patients were referred elsewhere for colonoscopy, primarily to 7 nearby locations (Figure).

**Figure:**
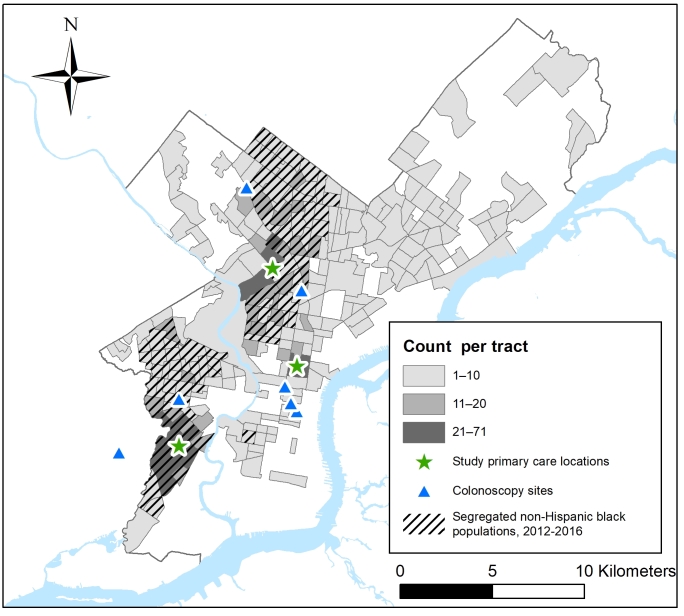
Number of Family Practice and Counseling Network (FPCN) patients in 2016 for whom colorectal cancer screening was recommended, by census tract of residence in Philadelphia, Pennsylvania; locations of FPCN clinic sites; locations of colonoscopy referral sites; and, for census tracts where patients resided, census tracts with higher levels of African American racial segregation relative to all Philadelphia census tracts. Six of the 7 facilities where FPCN refers patients for colonoscopy are located in Philadelphia, and 1 is located in an adjacent municipality, near the FPCN clinic in southwest Philadelphia.

**Cancer screening.** Screening status was based on information extracted from EHRs and criteria specified in HRSA’s Uniform Data System for annual reporting by FQHCs to the Bureau of Primary Health Care, which oversees the FQHC program (14). Patients for whom screening for colorectal cancer was recommended were men and women aged 51 to 74, and screening was classified as up to date if the patient had undergone a colonoscopy within 10 years before the patient’s last visit in 2016 or had completed a fecal immunochemical test within the preceding 12 months (14). Patients for whom cervical cancer screening was recommended were women aged 24 to 64, and screening was considered up to date if a Papanicolaou test had been completed within 24 months before the last visit in 2016 (14). These determinations were based on information extracted from patients’ EHRs in January 2018.

**Patient attributes.** We characterized each patient by using individual and census tract measures. Individual attributes were obtained from EHRs and included patients’ race/ethnicity, age as of January 2016, sex, insurance coverage (Medicare, Medicaid, private insurance, uninsured, unknown), and FPCN clinic site where they received primary care. We characterized the overall population in the census tract where each patient resided by using geographic indexes that reflect social determinants of health (15). Because of well-recognized associations between poverty or low educational attainment and a spectrum of adverse health outcomes (16), we used data from the American Community Survey (ACS) (17) 5-year estimates for 2012–2016 to describe the proportion of census tract residents living below the federally defined poverty threshold, the percentage of residents aged older than 25 who had graduated from high school, and the percentage of residents aged older than 25 who had received a bachelor’s degree. We also included an indicator of racial segregation, operationalized as the local G_i_* statistic (18). A census tract with a high proportion of African American residents surrounded by tracts with high proportions of African American residents, relative to all census tracts in Philadelphia, would have a high segregation index, expressed by using a *z* score (values >1.96 for an individual tract indicate a significant level of segregation). We used data from the 2012 Southeastern Pennsylvania Household Health Survey conducted by the Public Health Management Corporation (19) to define the percentage of census tract residents who perceived their neighborhoods as safe, on the basis of responses to 2 questions:

1. In the past month, did you not go someplace during the day because you felt you would not be safe?”
2. Is there a park or other outdoor space in your neighborhood that you’re comfortable visiting during the day?

Respondents who answered no to the first question and yes to the second were classified as perceiving that their neighborhoods were safe. A measure of perceived social capital from the 2014–2015 iteration of the same survey was based on responses to 3 questions and expressed on a scale of zero (never) to 3 (always):

1. How likely are people in your neighborhood willing to help their neighbors?
2. Most people in my neighborhood can be trusted.
3. I feel that I belong and am a part of my neighborhood.

For these 2 survey-based indexes, aggregate measures at the census tract level were created by using an empirical Bayes estimate, adjusted for individual-level sex and age and expressed as a weighted percentage for respondents (20,21). We obtained Philadelphia Police Department data on numbers of violent crimes (homicides, rapes, aggravated assaults) for 2016 from OpenDataPhilly (22) and calculated rates per 10,000 population based on census population estimate data from the ACS for 2012–2016. We examined correlations among census tract attributes, expressed as continuous variables, by using Pearson correlation *r* values.

**Geocoding.** We used ArcGIS 10.5 (Esri) with the Business Analyst 2016 Composite Address Locator (23) for automatic geocoding of patient addresses. For the 16,790 patients from Philadelphia and surrounding areas who were seen at an FPCN site in 2016, 93% of addresses were matched to street address that could be automatically geocoded. Following manual review of the remainder, 98% of addresses were geocoded. Patients for whom geocodes could not be obtained were excluded. Patients’ census tracts of residence were determined on the basis of the geocodes, and for each patient we added a variable that described the overall population for her or his census tract of residence for each of the geographic indexes.

**Statistical analysis.** We assessed univariate associations between patients’ individual and census tract characteristics and cancer screening proportions by using χ^2^ tests of contingency tables. We then used generalized estimating equations (GEEs) with an exchangeable correlation structure to calculate adjusted relative risks for associations between being unscreened and patient and neighborhood attributes. This approach allowed for simultaneous consideration of person-specific (age, sex, insurance status, clinic site) and group-level (characteristics shared by all patients living within a census tract) attributes. For all tabulations and comparisons of different groups of patients, reported proportions screened reflected the number screened among those for whom screening was recommended. To estimate risk ratios from the GEE models, we used the log binomial distribution method (24). For colorectal cancer, we first modeled the person-level attributes, including age group (50–59, 60–69, 70–75), sex (male, female), insurance status (Medicare, Medicaid, private insurance, uninsured/unspecified), and primary care site, absent the neighborhood attributes. To evaluate associations between the census tract measures and screening, we modeled each measure in separate GEE models adjusted for person-level characteristics. With the exception of racial segregation, which was considered as a dichotomous variable (<1.96, indicating lower levels of segregation, versus >1.96), all other census tract indicators were aggregated in ranges defined by terciles. For cervical cancer screening, we followed the same approach, except that sex was not included, and age groups were classified as 23 to 39, 40 to 49, and 50 to 64. All statistical tests were 2-tailed, and risk ratio estimates are shown with 95% confidence intervals (CIs). We considered *P* values < .05 (SAS, v 9.4 [SAS Institute Inc]) to be significant. The study was approved by the Drexel University Institutional Review Board.

## Results

Our study population consisted of 1,708 men and women for whom colorectal cancer screening was recommended and 4,995 women for whom cervical cancer screening was recommended (Table 1). The colorectal cancer screening group resided in 249 of Philadelphia’s 384 census tracts as defined in 2010, including 49 tracts with 10 or more study residents (Figure 1), and the cervical cancer screening group resided in 307 tracts, including 147 tracts with 10 or more study residents. Medicaid was the most common form of health insurance. Nearly half of those eligible for colorectal cancer screening and nearly two-thirds of those eligible for cervical cancer screening had Medicaid coverage (Table 1). Among those eligible for colorectal cancer screening, 63% were aged 50 to 59, 33% were 60 to 69, and 4% were 70 to 75. Among women eligible for cervical cancer screening, 65% were aged 23 to 39, 16% were 40 to 49, and 19% were 50 to 64. For both groups, approximately one-third of the population in census tracts where patients resided had household incomes below the federal poverty threshold, and fewer than 15% of those who were aged older than 25 had bachelor’s degrees.

**Table 1:** Characteristics of African American Patients Receiving Primary Care Services in a Network of Community Health Centers for Whom Colorectal Cancer Screening was Recommend (N=1,708) and for Whom Cervical Cancer Screening was Recommended (N=4,995), Philadelphia, Pennsylvania, 2016

Characteristic	Eligible for Colorectal Cancer Screening	Eligible for Cervical Cancer Screening
**Number of patients**	1,708	4,995
**Female, %**	67	100
**Mean age, years (SD)**	58.1 (6.0)	37.2 (11.4)
**Insurance coverage, %**		
Medicaid	48	64
Medicare	29	6
Private	16	20
Uninsured	7	10
Unknown	<1	<1
**Population attributes of patients’ census tracts of residence, median (interquartile range, q1–q3)^a^**		
Annual income below federal poverty level, %	33 (27–39)	33 (27–40)
Racial segregation index, *z* score^b^	2.5 (0.8–3.2)	2.6 (0.8–3.2)
Perceived their neighborhood as safe, %	83 (77–87)	83 (76–87)
Social capital index^c^	1.7 (1.6–1.9)	1.8 (1.6–1.9)
Violent crimes per 10,000 population	345 (236–435)	342 (236–440)
Completed high school, %	80 (76–85)	80 (75–85)
Have bachelor’s degree, %	12 (9–20)	13 (9–19)

a Median and interquartile (q1– q3) range of characteristics for populations residing in census tracts where study patients resided; number of census tracts represented in median values is the number of patients.

b Getis-Ord Gi* statistic, *z* scores >1.96 indicate significant racial segregation.

c 0 = lowest social capital, 3 = highest social capital.

Correlations among the census tract indexes varied from moderate to weak. For example, among the 249 census tracts where patients eligible for colorectal cancer screening resided, census tracts with higher levels of racial segregation had somewhat higher rates of violent crime (*r* = 0.34) and proportions living in poverty (*r* = 0.22) and lower proportions of adults with bachelor’s degrees (*r* = −0.38). Correlations between the segregation index and other measures were weaker, including perceived neighborhood safety (*r* = 0.04), social capital (*r* = −0.11), and proportions of adults who completed high school (*r* = 0.05).

**Colorectal cancer screening.** Among 1,708 men and women eligible for colorectal cancer screening, screening was up-to-date for 705 (41.3%). Among those who had been screened, 94% had undergone colonoscopy; others had a fecal immunochemical test. In univariate analyses, screening percentages were significantly higher (*P* < .05) among women than men, among those in older age groups, and among those who were insured than among those who were uninsured (Table 2). Significant differences in screening percentages were observed in univariate analyses for 2 census tract population attributes. Those living tracts with higher levels of racial segregation were less likely to be screened (37.4%) than those living in tracts with lower levels of racial segregation (47.7%), and screening percentages were highest among patients living in census tracts where greater proportions of residents had bachelor’s degrees (Table 2).

**Table 2:** Primary Care Patients (N = 705) Up to Date With Colorectal Cancer Screening in 2016, by Individual-Level Attributes and Attributes of Populations in Their Census Tracts of Residence, Philadelphia, Pennsylvania, 2016

Patient and Census Tract Attributes	Percentage Screened (Univariate Analysis)	aRR^a^ of Being Unscreened (95% Confidence Interval)
**All eligible patients, %**	41.3	NA
**Age, y**		
50–59	36.5^b^	1.27 (0.97–1.67)
60–69	48.8	1.11 (0.85–1.45)
70–75	55.4	Reference
**Sex**		
Male	37.3^b^	Reference
Female	43.2	0.88 (0.82–0.93)^c^
**Insurance coverage**		
Medicaid	39.3^b^	Reference
Medicare	50.2	0.90 (0.80–1.00)
Private	41.8	0.97 (0.86–1.09)
Uninsured/unknown	19.3	1.30 (1.22–1.39)^c^
**Population Attributes of Patients’ Census Tracts of Residence^d^**		
**Racial segregation index, *z* score^e^**		
≤1.96	47.7^b^	0.91 (0.84–0.99)^c^
>1.96	37.4	Reference
**Annual income below federal poverty level, %**		
1–28	39.6	1.06 (0.94–1.21)
28–36	44.5	1.00 (0.87–1.14)
36–67	39.8	Reference
**Violent crimes per 10,000 population**		
29–289	41.1	1.04 (0.94–1.14)^f^
290–408	40.4	1.09 (0.99–1.19)^f^
409–1,077	42.4	Reference
**Neighborhood perceived as safe, %**		
29–80	43.4	0.91 (0.80–1.04)
80–86	37.7	1.03 (0.91–1.16)
86–95	43.2	Reference
**Social capital index, possible range 0–3**		
1.04–1.67	43.1	0.93 (0.82–1.04)
1.67–1.83	37.7	0.99 (0.87–1.13)
1.83–2.38	43.0	Reference
**Completed high school, %**		
43–77	41.4	0.92 (0.81–1.04)
77–83	39.5	1.00 (0.88–1.12)
83–100	43.0	Reference
**Completed bachelor’s degree, %**		
1–10	38.8^b^	0.97 (0.84–1.11)
10–17	37.5	0.93 (0.81–1.06)
17–79	47.6	Reference

Abbreviation: NA, not applicable.

a aRR = Adjusted estimate of relative risk of being unscreened. For individual-level patient attributes, values are shown for models that did not include census tract attributes; aRR values for these attributes were similar in all models that included census tract attributes, and aRR values with confidence intervals that excluded 1.0 were the same.

b *P* < .05 for contingency table with 2 to 4 rows (depending on variable) and 2 columns (screened versus unscreened).

c *P* < .05, 2-tailed.

d These measures describe attributes of the population in each patient’s census tract of residence. Categories are terciles for all measures, except for racial segregation index. Tercile boundaries for census tract attributes might appear to overlap because of rounding.

e A *z* score of >1.96 indicates significant racial segregation in census tract where patient resides.

f Full model including all individual-level variables did not converge; aRR and confidence intervals are shown for model that excludes clinic site.

In the final GEE models, individual-level attributes that remained significantly associated with being unscreened were male sex and being uninsured (Table 2). Among census tract attributes, only racial segregation remained significant, with an adjusted relative risk of being unscreened of 0.91 (95% confidence interval, 0.84– 0.99) for those living in more versus less segregated tracts (ie, those living in less segregated neighborhoods were nearly 10% more likely to be screened than those living in more segregated neighborhoods). The association between racial segregation and screening persisted with addition of census tract poverty levels to the model and was not modified by tract-level educational attainment at the bachelor’s degree level.

### Cervical cancer screening

Among 4,995 women eligible for cervical cancer screening, 3,760 (75.3%) were screened. In univariate analyses, screening percentages were significantly higher among younger than older women and among those with insurance than those who were uninsured (Table 3). We observed significant differences in screening percentages in univariate analyses for 3 census tract population attributes. Those living in tracts with higher levels of racial segregation were less likely to be screened than those living in tracts with lower levels of racial segregation; those living in census tracts with the lowest poverty levels had lower screening percentages than those living in tercile groups with higher levels of poverty; and those living in tracts with the 2 highest levels of perceived neighborhood safety had lower screening percentages than those living in tracts that residents perceive as the least safe.

**Table 3:** Primary Care Patients (3,760) Up to Date With Cervical Cancer Screening in 2016, by Individual-Level Attributes and Attributes of Populations in Their Census Tracts of Residence, Philadelphia, Pennsylvania, 2016

Patient and Census Tract Attributes	Percentage Screened (Univariate Analysis)	aRR of Being Unscreened (95% Confidence Interval)^a^
**All eligible patients**	75.3	NA
**Age, y**		
23–39	79.9^b^	0.52 (0.47–0.58)^c^
40–49	73.9	0.68 (0.59–0.77)^c^
50–64	60.9	Reference
**Insurance coverage**		
Medicaid	76.1^b^	Reference
Medicare	67.9	0.99 (0.83–1.19)
Private	80.0	0.84 (0.73–0.97)^c^
Uninsured/unknown	64.5	1.39 (1.22–1.59)^c^
**Population Attributes of Patients’ Census Tracts of Residence^d^**		
**Racial segregation index,* z* score^e^**		
≤1.96	78.3^b^	0.97 (0.86–1.10)
>1.96	73.5	Reference
**Annual income below federal poverty level, %**		
3–28	72.9^b^	1.06 (0.94–1.21)
28–37	77.0	1.00 (0.87–1.14)
37-67	76.0	Reference
**Violent crimes per 10,000 population**		
24–283	75.9	1.01 (0.89–1.15)
285–408	74.8	0.95 (0.85–1.07)
409–877	75.2	Reference
**Neighborhood perceived as safe, % **		
29–79	78.4^b^	0.91 (0.80–1.04)
79–85	72.9	1.03 (0.91–1.16)
86–95	74.5	Reference
**Social capital index, possible range 0–3**		
1.04–1.67	75.7	0.93 (0.82–1.04)
1.67–1.83	74.2	0.99 (0.87–1.13)
1.83–2.35	75.6	Reference
**Completed high school, % **		
43–76	76.4	0.92 (0.81–1.04)
77–83	74.6	1.00 (0.88–1.12)
83–100	74.9	Reference
**Completed a bachelor’s degree, %**		
1–10	74.5	0.97 (0.84–1.11)
10–15	74.9	0.93 (0.81–1.06)
15–88	76.5	Reference

Abbreviation: NA, not applicable.

a aRR = Adjusted estimate of relative risk of being unscreened. For individual-level patient attributes, values are shown for models that did not include census tract attributes; aRR values for these attributes were similar in all models that included census tract attributes, and aRR values with confidence intervals that exclude 1.0 were the same.

b *P* < .05 for contingency table with 2 to 4 rows (depending on variable) and 2 columns (screened versus unscreened).

c *P* < .05, 2-tailed.

d These measures describe attributes of the population in each patient’s census tract of residence. Categories are terciles for all measures, except for racial segregation index. Tercile boundaries for census tract attributes might appear to overlap because of rounding.

e A *z* score of >1.96 indicates significant racial segregation in census tract where patient resides.

In the final GEE models, individual-level attributes that remained significantly associated with differences in being unscreened were age and insurance status (Table 3). After controlling for individual-level attributes, none of the census tract attributes retained an independent association with screening percentages (Table 3).

## Discussion

In our study group of African American patients residing in Philadelphia neighborhoods and receiving primary care in an FQHC network, 41% and 73%, respectively, had been screened for colorectal and cervical cancer per national guidelines. This compares with self-reported rates of colorectal and cervical cancer screening of 68% and 77% statewide in Pennsylvania in 2016 and to 2020 national targets of 71% (colorectal cancer) and 93% (cervical cancer) (25,26). In comparing patients who had and had not been screened for colorectal cancer, those living in census tracts with relatively high levels of racial segregation were less likely to be screened than those living in less segregated neighborhoods. Other population-level attributes in census tracts where patients resided were not associated with screening levels for either cancer, after taking into account their site of care and personal characteristics, including insurance coverage.

The observation that racial segregation was associated with lower rates of screening for colorectal but not cervical cancer likely reflects differences between the screening procedures, attitudes regarding colorectal cancer screening, and consequences of racial segregation. Although cervical cancer screening is integrated into routine primary care and provided on site at FPCN clinics, obtaining a colonoscopy requires referral to another facility, scheduling, dedication of time and effort on the day before the procedure to prepare, dedication of a day for the procedure itself, which requires general anesthesia, and being accompanied by someone who can assure safe transport afterwards. Lower rates of colonoscopy among African American populations relative to white populations are associated with reluctance concerning the procedure and under-recognition of both the risk of colorectal cancer and the value of colonoscopy (27). Racial segregation is well-recognized as a contributor to health disparities and is associated with physical and social attributes of neighborhoods that adversely affect health and health-related behaviors and that stress individuals and families (28,29), which altogether likely aggravate colonoscopy reluctance and complicate completion of a colonoscopy referral.

Our investigation was prompted by an interest in assessing the utility of geographically defined social health determinants as a complement to individual, self-reported measures. Associations between screening completion and social health determinants at the neighborhood population level might differ from associations between screening completion and individuals’ assessments of their neighborhoods (10). For example, we observed that a population-level index of social capital based on aggregated responses to a community health survey was not associated with variations in cervical or colorectal cancer screening. In contrast, Leader and Michael studied self-reported screening rates in Philadelphia by using data from the same survey and observed that individual-level responses to questions regarding neighborhood social capital were associated with screening rates, and greater social capital was more strongly associated with higher colorectal than cervical cancer screening (9). This suggests that population-level indexes that describe the neighborhoods where patients live are an imperfect proxy for obtaining information about social determinants directly from patients. Nonetheless, when attributes of the places where patients live are found to be associated with variations in cancer screening, this knowledge might be useful in identifying patients most at risk for not completing recommended screenings.

This study has several limitations. We did not calculate patients’ ages at the time of care on the basis of their dates of birth; rather, to protect confidentiality, we obtained data for patients’ ages at the start of the study period. This might have led to the misclassification of age relative to age-specific screening recommendations over the 1-year study interval. Classifications of whether screening was up to date were based on routine HRSA reporting criteria and did not take into account patient attributes such as family history or prior screening results that might call for more frequent screening or the potential effect of applying more nuanced screening criteria, such as those established by the American Cancer Society (30). We were not able to consider personal income levels as a measure of individual-level poverty because income data were not available for nearly a third of patients and because of the inability to relate income levels to household size. Our study focused on African American patients receiving primary care at an FQHC network in Philadelphia, and our findings might not be generalizable to patients served by other FQHCs, either in Philadelphia or other urban areas.

In conclusion, among African American patients receiving primary care within an FQHC network, those living in more racially segregated neighborhoods were less likely to be screened for colorectal cancer. Other neighborhood-level population attributes that reflect social determinants of health were not associated with variations in cervical or colorectal cancer screening. This association with racial segregation is consistent with recognized adverse effects of segregation on health-related behaviors (28,29). Although our findings suggest that neighborhood attributes are unlikely to be an effective proxy for direct inquiries of patients regarding social determinants of health, they might be useful in targeting and informing community outreach efforts to improve colorectal cancer screening.
